# Functional maturation of nasal mucosa: role of secretory immunoglobulin A (SIgA)

**DOI:** 10.1186/2049-6958-8-46

**Published:** 2013-07-18

**Authors:** Luisa Bellussi, Jacopo Cambi, Desiderio Passali

**Affiliations:** 1 ENT Department, University of Siena, Siena, Italy

**Keywords:** Nasal mucosa maturation, Secretory immunoglobulin A, SIgA, Upper respiratory tract infection

## Abstract

Secretory IgA (SIgA) plays an important role in defending the mucous membranes of the upper respiratory airways from common infection. Studying and comparing the values and the daily variation of SIgA in nasal secretion could explain the largest number of upper respiratory infection, especially in children.

Moreover, the ELISA dosage of SIgA in nasal secretion, sampled by cotton swabs positioned between septum and middle turbinate for 20 minutes every 4 hours 5 times in a day, can be easily performed and shows significant differences between the healthy child and the healthy adult.

Nasal secretion SIgA mean value is lower in the healthy child than in the healthy adult. Circadian variation for healthy child showed the highest value at 7.00 a.m., while in adult the highest value was at 4.00 a.m.

These knowledge on SIgA may help to explain the highest number of upper airway infection during childhood and clarify the physiological cycle of production. Thus, in performing a SIgA dosage the time of sample must be considered and preferably it should be made at a standardized time of the day.

## Background

In children upper respiratory tract infection (URTI) is one of the most frequent reasons for physician visits. Nasal respiratory function grows up gradually in childhood together with nasal and sinus anatomical structure and immunological defences. Only after puberty the nose reaches an anatomical and functional maturation and together with pharynx and Eustachian tubes constitute the rhino-pharyngo-tubal unit [1].

Secretory Immunoglobulins A (SIgA) play a key role in the elaboration of the immunological response at the level of the upper respiratory airway, challenging allergens or pathogenic micro organisms. In fact, distributed on the mucous film lining the respiratory epithelium, SIgA neutralize viruses and bacterial surface antigens, favouring their phagocytosis.

The study of the circadian pattern variation and the values of SIgA in nasal secretion of healthy child may explain the greater number of URTI during childhood compared with adult healthy pattern.

## Main text

In childhood the incomplete development of the nasal macroscopic morphology is coupled with an immaturity at microscopic level so that constitutional or acquired defensive factor are inadequate.

The IgA secreted across the mucosa prevents microbial binding to epithelial cells in the digestive and respiratory tracts. It exists in two forms, that differ in the structure: IgA1 is found in serum and produced by bone marrow B cells, whereas IgA2 is made by B cells located in the mucosa and has been found to be secreted into colostrum, maternal milk, tears and saliva. SIgA is composed of two immunoglobulin A molecules, which are joined by a J-protein and a secretory component. After binding, the SIgA is transported across the cell and excreted by exocytosis [2].

SIgA can be collected in saliva, but the salivary flow rate and alpha-amylase activity, and the autonomic nervous system activation play a determinant role on the SIgA synthesis . On the contrary, in nasal secretion these variables are absent, such as the assay is more reproducible and reliable although more cumbersome to implement [3].

The role of SIgA has long been studied in adults. In chronic rhinosinusitis specific SIgA antibody activity to the bacterial M protein plays a significant role in preventing or blocking the adhesion of bacteria [4]. A greater amount of bacterial IgA1 protease causes a confined impairment of the nasal mucosal immune barrier and may be a primary event in the pathogenesis of inflammatory respiratory disease [5].

In children there is no significant difference in IgG, IgA and IgE in nasal secretion between healthy subjects and those with recurrent upper airway infection, and only albumin values increase during infection [6].

Nasal secretions could be sampled by the insertion between the middle turbinate and the septum of swabs of cotton which are removed after 20 minutes and immediately squeezed out. Subjects must be instructed to leaning backwards the head in order to reduce any contamination with tears as any secretion from the nasolacrimal duct would run along the nasal floor. To determinate the circadian pattern of SIgA the samples were collected at fixed time points within 8 consecutive 4-hours periods starting at 7.00 a.m., stored at −18°C until shipped to laboratory for SIgA dosage with ELISA kit for human SIgA according to the “sandwich” principle. To overcome the daily variability samples can be collected for 3 consecutive days and after 2 weeks [7].

In child allergies, use of antibiotics or presence of respiratory infection 15 days before the examination, made to confirm the diagnosis or the suspicion of immunodeficiency disease or ciliary dyskinesia, could change the effective value of SIgA.

Nasal secretion SIgA mean value was lower in the healthy child than in the healthy adult. Circadian pattern for healthy child shows the highest value at 7.00 a.m., while in adult the highest value was at 04.00 a.m. and the curve remains upper the child value for all the day. In child after early morning the diurnal variations in SIgA are characterized by a sudden fall in the late morning (10.00 a.m. - 1.00 p.m.) followed by a small peak at 4.00 p.m. and a flat trend until the next morning. In adult the SIgA course is more regular, after the peak there is a gradual decrease until the lower level at 1.00 p.m. that is followed by a steady increase in the afternoon [8]. These data were obtained from 10 healthy children (5 males; age 4,7 ± 1,2 years) and 10 healthy adults (6 males; age 41,3 ± 13,2 years).

## Discussion

The childhood immunological immaturity is evident at the level of the nasal mucosa where a complete cell-mediated reactivity is coupled with an inadequate humoral immunity.

If at birth a sufficient level of antibodies is guaranteed by the transplacental passage from mother to newborn, in the first months of life the mother's milk ensures a sufficient amount of SIgA to reinforce the defences of the mucosa, such as the nasal mucosa, which is at the forefront in the interaction with the outside environment. After weaning, a period of immunological difficulty begins because still immature mucociliary transport and nasal cycle are added to low levels of SIgA.

Human processes and functions are structured in space and time, biological rhythms could have short-, intermediate-, and long-period fluctuations. Circadian clocks are located in the suprachiasmatic nuclei and are perhaps the best characterized biological oscillators; they influence physiology and behaviour, including sleep-wake cycles, cardiovascular activity, endocrinology, body temperature [9]. The circadian (24-h) time organization has been most studied and shows great significance in clinical medicine. The phase and amplitude of circadian rhythms contribute to physiological manifestation like nasal cycle and mucociliary transport and determinate the severity of symptoms in chronic diseases like allergic rhinitis. Furthermore, body rhythms can considerably influence responses of patients to diagnostic tests [10].

In allergic rhinitis the major symptoms of runny and blocked nose worsen upon waking up in the morning, because the circadian variation in nasal reactivity is higher at 6.00 a.m. and influences also the activity (higher levels of inflammatory cytokines) of eosinophils and basophilic cells in the nasal mucosa [11].

The nasal muco-ciliary transport has a circadian variation too (is faster in the evening) and varies with age [12]. In childhood there is an insufficient number of ciliate cells and mucus-producing elements at tuba and rhinopharyngeal level so that mucociliary transport is immature and the periodic fluctuations in turgidity of the cavernous tissue of turbinates are not alternate but synchronous on both sides [1].

The main factor that influences these characteristic changes of childhood and thereforealso the SIgA is that the circadian rhythm is not fully developed yet as in the adult. Only during adolescence the SIgA circadian secretion pattern reaches the adult values.

## Conclusions

The knowledge that in children there is a lack of SIgA may help to explain the highest number of URTI during childhood and promotes further studies about upper respiratory immune system maturation. Furthermore, the cycle of SIgA production must be considered and the exact hour of sample must be reported and evaluated during the analysis of results. In fact, if a measurement of SIgA is performed at different hours, it will show different values due to the circadian cycle of production. It is therefore essential to perform the SIgA dosage always at the same time and only in this way it will be possible to avoid a wrong attribution to pathological situations for apparently high or low values.

## Abbreviations

SIgA: Secretory immunoglobulin A; URTI: Upper respiratory tract infection.

## Competing interests

There isn’t any financial research support and conflict of interest for this study. All authors have not any disclosure.

## Authors’ contributions

LB, JC, DP: Formulating the hypothesis and writing of the manuscript; DP: Review of the manuscript and data analysis. All authors read and approved the final manuscript.

## Acknowledgements

Special thanks to Prof. Filippo Ricciotti chairman of "Unità di Patologia Clinica" of Marino Hospital - Roma.
